# Systems biology in the era of AI: “winter” or “evolution”?

**DOI:** 10.3389/fsysb.2026.1818525

**Published:** 2026-03-24

**Authors:** Mohamed Helmy, Kumar Selvarajoo

**Affiliations:** 1 Vaccine and Infectious Diseases Organization (VIDO), University of Saskatchewan, Saskatoon, SK, Canada; 2 Vaccinology and Immunotherapeutics Program, School of Public Health, University of Saskatchewan, Saskatoon, SK, Canada; 3 Department of Computer Science, University of Saskatchewan, Saskatoon, SK, Canada; 4 Department of Biochemistry, Microbiology and Immunology, College of Medicine, University of Saskatchewan, Saskatoon, SK, Canada; 5 Department of Computer Science, Idaho State University, Pocatello, ID, United States; 6 Bioinformatics Institute (BII), Agency for Science, Technology and Research (A*STAR), Singapore, Singapore; 7 Synthetic Biology Translational Research Program, Yong Loo Lin School of Medicine, National University of Singapore (NUS), Singapore, Singapore; 8 Synthetic Biology for Clinical and Technological Innovation (SynCTI), National University of Singapore (NUS), Singapore, Singapore; 9 School of Biological Sciences, Nanyang Technological University (NTU), Singapore, Singapore

**Keywords:** artificial intelligence - AI, bioinformatics, data science, modelling, systems biology

## Introduction

The sequencing and release of the draft human genome in June 2000 generated immense excitement, with expectations of a “quantum leap” in understanding molecular biology and human disease (Lander et al., 2001). Around the same period, *systems biology* emerged as a multidisciplinary effort uniting physics, mathematics, bioinformatics, and computational modeling. The field promised transformative advances in deciphering complex biological systems, their emergent behaviors, and potential therapeutic targets (Hasty et al., 2001; Kitano, 2002). This optimism spurred the launch of dedicated journals such as *Molecular Systems Biology* (EMBO), *BMC Systems Biology*, *PLOS Computational Biology*, and *npj Systems Biology and Applications*.

Systems biology indeed enabled discoveries unattainable through reductionist approaches alone. For example, dynamic models of inflammatory responses offered mechanistic insights and identified regulators that were later experimentally validated (Hayashi et al., 2013; Caldwell et al., 2014). Its core strength lies in moving from descriptive correlations to causal explanations, incorporating nonlinearity, feedback, and emergent properties (Kitano, 2001).

Yet despite its promise, the field has faced hurdles. Building dynamic or kinetic models of biological networks, intended to predict changes in molecular concentrations over time, has proven slow. The difficulty stems from quantifying large numbers of model parameters, limited availability of time-resolved data, and the heavy mathematical demands of parameter estimation and optimization.

As with stem cell biology and gene therapy, systems biology has undergone recalibration: early enthusiasm outpaced translation, and breakthroughs in curing major diseases such as cancer and diabetes remain elusive (Daley, 2012; Kaemmerer, 2018). Progress now depends on new technologies, particularly automated and scalable modeling approaches.

## The rise of AI in biomedicine

In recent years, artificial intelligence (AI), especially generative models, has been widely adopted to analyze multi-omics datasets, generate artificial data, and even build *in silico* cancer genomes (Heydari et al., 2022; Díaz-Navarro et al., 2025; Woerner et al., 2025). AI excels at uncovering hidden patterns in vast datasets, often at scales far beyond human capacity. However, these models remain largely predictive “black boxes,” powerful for classification and pattern recognition but offering little mechanistic explanation (Helmy et al., 2020).

Enthusiasm has nonetheless soared. Global investment in AI-driven biomedical research has increased dramatically (Chibambo, 2025; Clarcke, 2025; Greenacre, 2025; MDDI, 2025; NIH, 2025). This raises a philosophical question: should biomedicine prioritize rapid prediction without explanation, or aim for explanations that enable reliable, reproducible prediction?

## Is systems biology facing a winter?

**FIGURE 1:**
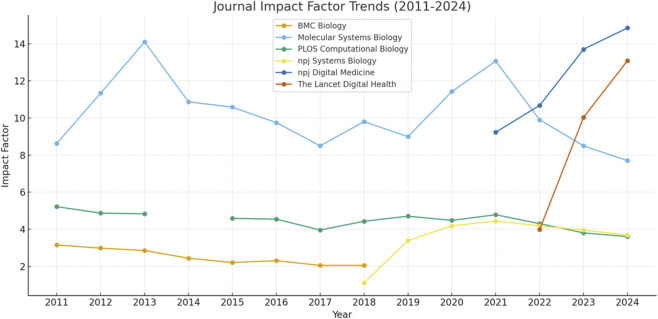
The impact factor of Systems Biology and Biomedical AI journals since 2011 (retrieved from journal websites and Web of Science). Gradual decline in BMC Systems Biology (ceased in 2019) and PLOS Computational Biology, whereas a surge in npj Digital Medicine and The Lancet Digital Medicine.

Foundational systems biology research now faces critical challenges. The closure of *BMC Systems Biology* in 2019, together with declining impact factors of related journals, points to reduced visibility and engagement in the field. Although impact factor is an imperfect metric, it reflects average citation activity and is therefore a reasonable proxy for community interest in this context, especially when contrasted with the rapid rise of AI-focused outlets such as *npj Digital Medicine* and *The Lancet Digital Health* (Figure 1). This shift reflects a redistribution of attention and resources. Yet such rapid swings risk undervaluing the mechanistic insights systems biology uniquely provides. If training pipelines focus too heavily on AI, future scientists may excel at algorithmic manipulation but lack the ability to design or test mechanistic hypotheses, skills that, once lost, are slow to rebuild (Pellegrina and Helmy, 2025).

Are we witnessing the first “systems biology winter,” akin to the AI winters where unmet expectations deflated momentum (Toosi et al., 2021)? Or is this a natural stage of maturation? As shown in Figure 1, based on journal impact, systems biology appears to have plateaued or gradually declining, while AI is experiencing exponential growth.

## Integration rather than competition

A more constructive interpretation is that systems biology is evolving into a complementary partner to AI. Rather than competing, the two can be integrated.

Systems biology provides mechanistic guardrails, ensuring AI predictions remain biologically plausible. AI, in turn, accelerates discovery by scanning high-dimensional datasets for candidate interactions (Helmy et al., 2020; Yeo and Selvarajoo, 2022). This synergy aligns with the vision of *Digital Twins*, virtual representations of patients that combine mechanistic physiology with AI-driven personalization to predict disease trajectories and treatment responses (Laubenbacher et al., 2022; Helmy et al., 2024).

Recent advances in deep learning for causal network inference (Lagemann et al., 2023; Van Hilten et al., 2024) highlight both promise and limitations: predictions are largely static and often inaccurate (Ahlmann-Eltze et al., 2025). Embedding systems biology models within AI frameworks could provide the missing temporal and mechanistic structure.

Integration also addresses ethical imperatives. Black-box predictions in medicine risk misleading clinical practice and policy if not grounded in mechanistic understanding (Budhkar et al., 2025). As argued in emerging frameworks for responsible AI (Helmy et al., 2025), interpretability and reproducibility are ethical requirements, not optional features.

## A roadmap for the future

To accelerate integration, we propose a staged roadmap:

- Short-term (0–3 years):
  ○ Embed dynamic systems models into AI workflows for temporal plausibility.
  ○ Establish benchmarking standards where AI predictions are cross-validated with mechanistic models.
- Mid-term (3–7 years):
  ○ Funding agencies and journals prioritize integrative projects.
  ○ Develop collaborative platforms linking systems biology datasets with AI pipelines.
- Long-term (7+ years):
  ○ Redesign training programs to blend algorithmic literacy with mechanistic reasoning.
  ○ Foster translational pipelines where AI-driven discoveries are grounded in systems biology for clinical application.

## Conclusion

While systems biology no longer commands the excitement of the early 2000s, it remains indispensable. Its focus on causality, feedback, and emergent properties ensures biomedical discoveries are mechanistically grounded and reproducible. AI, by contrast, provides unprecedented predictive power but risks fragility without biological grounding. The future lies in integration. By combining AI’s scale and speed with systems biology’s explanatory depth, the biomedical sciences can achieve robust prediction and understanding. Whether systems biology faces a “winter” or an evolutionary transformation will depend on whether the community embraces this hybrid vision. With thoughtful policy, training, and interdisciplinary collaboration, systems biology can rise again, not as AI’s competitor, but as its essential partner.
